# Mapping Knowledge Gaps of Mozambique’s Terrestrial Mammals

**DOI:** 10.1038/s41598-019-54590-4

**Published:** 2019-12-03

**Authors:** Isabel Queirós Neves, Maria da Luz Mathias, Cristiane Bastos-Silveira

**Affiliations:** 1 Museu Nacional de História Natural e da Ciência, Universidade de Lisboa, Rua da Escola Politécnica 58, 1250-102 Lisbon, Portugal; 2 Departamento de Biologia Animal, Faculdade de Ciências da Universidade de Lisboa (FCUL), Campo Grande, 1749-016 Lisbon, Portugal; 3 Centro de Estudos de Ambiente e Mar (CESAM), Faculdade de Ciências da Universidade de Lisboa (FCUL), Campo Grande, 1749-016 Lisbon, Portugal; 4 Natural History and Systematics Research Group - Centre for Ecology, Evolution and Environmental Changes (CE3C), FCUL, Campo Grande, Lisbon, 1749-016 Portugal

**Keywords:** Biodiversity, Conservation biology

## Abstract

A valuable strategy to support conservation planning is to assess knowledge gaps regarding primary species occurrence data to identify and select areas for future biodiversity surveys. Currently, increasing accessibility to these data allows a cost-effective method for boosting knowledge about a country’s biodiversity. For understudied countries where the lack of resources for conservation is more pronounced to resort to primary biodiversity data can be especially beneficial. Here, using a primary species occurrence dataset, we assessed and mapped Mozambique’s knowledge gaps regarding terrestrial mammal species by identifying areas that are geographically distant and environmentally different from well-known sites. By comparing gaps from old and recent primary species occurrence data, we identified: (i) gaps of knowledge over time, (ii) the lesser-known taxa, and (iii) areas with potential for spatiotemporal studies. Our results show that the inventory of Mozambique’s mammal fauna is near-complete in less than 5% of the territory, with broad areas of the country poorly sampled or not sampled at all. The knowledge gap areas are mostly associated with two ecoregions. The provinces lacking documentation coincide with areas over-explored for natural resources, and many such sites may never be documented. It is our understanding that by prioritising the survey of the knowledge-gap areas will likely produce new records for the country and, continuing the study of the well-known regions will guarantee their potential use for spatiotemporal studies. The implemented approach to assess the knowledge gaps from primary species occurrence data proved to be a powerful strategy to generate information that is essential to species conservation and management plan. However, we are aware that the impact of digital and openly available data depends mostly on its completeness and accuracy, and thus we encourage action from the scientific community and government authorities to support and promote data mobilisation.

## Introduction

Effective conservation planning relies on insightful knowledge and data acquisition about species occurrence and distribution^1^. Primary species-occurrence data across dispersed data sources can be a cost-effective resource for boosting knowledge about a country’s biodiversity^2^. Particularly for poorly documented countries filling data gaps is crucial for new and broad insights for biodiversity research and conservation. Research-neglected regions, which lack quality information, coincide mainly with the species-rich and developing nations^3^.

**Figure 1:**
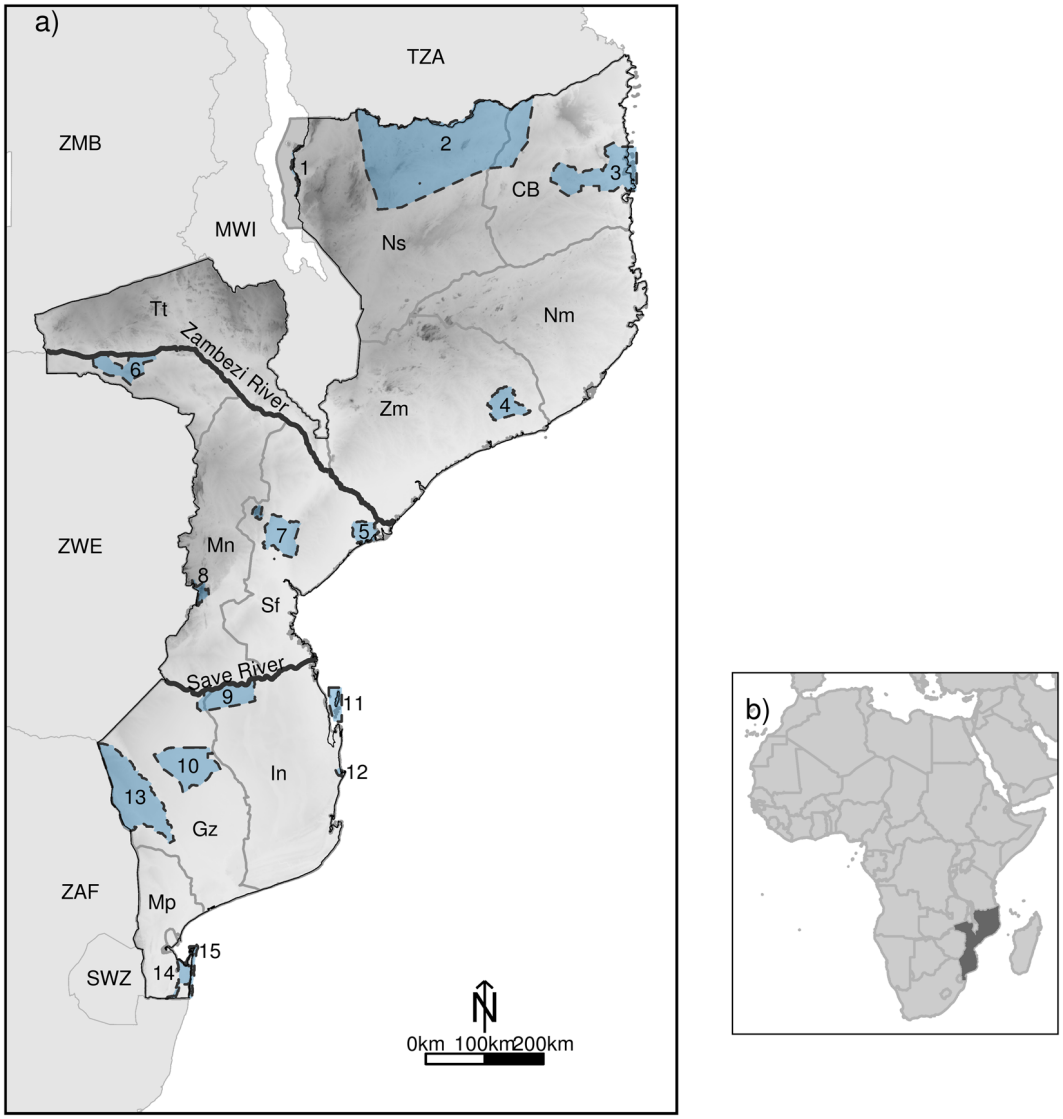
(**a**) Map of Mozambique, with the indication of the protected areas and the rivers that divide the country into three major biogeographical regions (dark line): North Mozambique, Central Mozambique and South Mozambique; and (**b**) Inset with the location of the Republic of Mozambique in the African continent. Mozambique is surrounded by six neighbour countries indicated in the figure by a three-letter code: Tanzania (TZA), Malawi (MWI), Zambia (ZMB), Zimbabwe (ZWE), South Africa (ZAF), and Swaziland (SWZ). Mozambique’s provinces are identified with a 2-letter code: Niassa (Ns); Cabo Delgado (CB), Nampula (Nm), Zambézia (Zm), Tete (Tt), Manica (Mn), Sofala (Sf), Inhambane (In), Gaza (Gz), Maputo (Mp). The country’s protected areas are indicated with a number: 1. Lake Niassa partial reserve, 2. Niassa national reserve, 3. Quirimbas national park, 4. Gilé national reserve, 5. Marromeu national reserve, 6. Mágoè national park, 7. Gorongosa national park, 8. Chimanimani national reserve, 9. Zinave national park, 10. Banhine national park, 11. Bazaruto national park, 12. Pomene national reserve, 13. Limpopo national park, 14. Maputo special reserve, 15 – Ponta do Ouro national reserve. Protected areas shapefile was downloaded from Biofund platform of conservation areas (http://www.biofund.org.mz/en/database/platform-of-the-conservation-areas/).

Mozambique, in southeastern Africa (Fig. 1), holds a rich, but poorly documented, biodiversity^4,5^. The country’s political instability from 1964 to 1992, due to a long period of war, led to species extirpations and irregular migrations, degradation of important ecosystems and a scarcity of biodiversity studies^6^. Despite recent monitoring efforts, mainly in protected areas, and contributions that greatly improved current knowledge on several taxonomic groups, there remains a significant lack of knowledge regarding the occurrence and distribution of most Mozambican species^4,7^.

The most recent inventory of terrestrial mammals from Mozambique, which was based on primary species occurrence data from several sources, reported a total of 217 species for the country^7^. The authors detected a taxonomic bias in the data towards large mammal groups, with only half of the small mammal species recorded during the last two decades.

The extent of biases in primary species occurrence data often results in over-representation of particular species or localities, concealing the real patterns of species distribution^1,8–13^. These biases are frequently a consequence of the historical, scientific interest in some areas, such as protected areas, and the inaccessibility of the other regions far from roads or river networks^14^. Biased data, in turn, weakens the utility of the compiled species distribution maps, especially for the species rich countries in the countries^15,16^. In the last decade, to overcome these data challenges, several authors made an effort to develop tools to analyse and describe biases and knowledge gaps in primary species-occurrence data^2,17–20^. The premise is that knowledge of data biases and uncertainty is fundamental to interpreting the mapped species distribution adequately^9,21,22^.

Furthermore, the assesment of knowledge gaps from primary species occurrence data to select areas for future biodiversity data to select areas for future biodiversity surveys is a useful strategy to support conservation planning. The evaluation of gaps from primary data can be achieved by calculating inventory completeness (i.e. the fraction of species in a given location that has been sampled) and by selecting areas with insufficient sampling and that are geographically distant and environmentally different from the well-known areas^2,23,24^. For understudied countries where the lack of resources for conservation is pronounced^25^, this strategy is particularly beneficial as survey effort focused on areas less visited and unique will likely produce new records or new species^2,11^.

In the present work, we assessed knowledge gaps on terrestrial mammal species from Mozambique aiming to provide baseline information for conservation planning. To achieve this goal, we evaluated: (i) the spatial and environmental biases of the mammal inventory in Mozambique; (ii) cell-wide inventory completeness, and (iii) sites with incomplete sampling that are geographically and environmentally unique. The approach here followed, which can be applied to other understudied countries, has the potential to generate reliable biodiversity information that can contribute towards effective conservation and management planning.

## Results

### Data description

Our analysis was based on data from the most recent inventory of terrestrial mammals reported for Mozambique^7^. This work compiled primary species occurrence data from several digital and non-digital sources of primary species occurrence data, namely: online platforms, museum collections, survey reports and scientific literature. The underlying data that supports the validated species checklist comprises a total of 14981 records, dated from 1842 to 2018, representing 217 mammal species.

Here, we reduced the inventory’s species occurrence data to unique records to avoid duplication of information, and records were aggregated to a 0.25° resolution country-wide grid (see Material and methods). The reduction of species occurrence data to unique records resulted in 14201 records of 215 species. Two species did not pass the data reduction process because the corresponding records did not contain enough information to be allocated to the country grid. These species were the bats *Chaerephon nigeriae* Thomas, 1913 and *Rhinolophus rhodesiae* Roberts, 1946.

**Figure 2:**
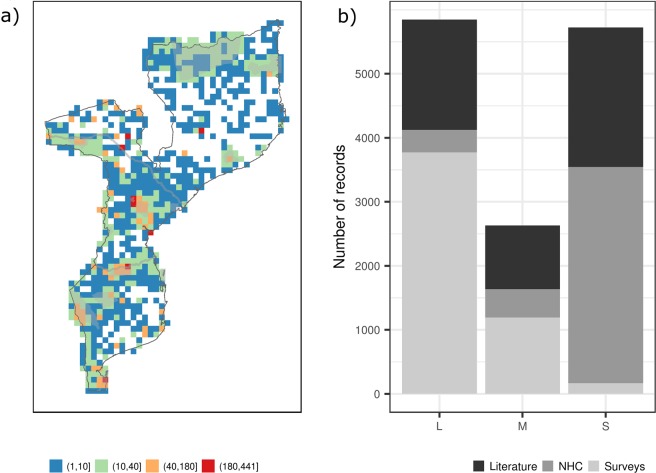
The number of records of Mozambique’s terrestrial mammals. (**a**) Visualisation of the number of unique records across Mozambique based on a 0.25° resolution grid. (**b**) Bars are showing the number of unique records per mammal group and the contribution of data sources. Mammal groups in the x-axis are identified by an “L” for large mammals, “M” for medium mammals, and “S” for small mammals.

The total number of grid cells across Mozambique that held mammal records was 1014, corresponding to 83.3% of the country (Fig. 2; Table 1). Most of the inventory data (almost 60%) were collected before the year 2000 (“old data”). These data correspond to a total of 204 species and are distributed across almost 68% of the country’s territory. The primary sources of these old data were literature (56.7%) and natural history collections (43.2%). On the other hand, records collected after the year 2000 (“recent data”) included 156 species and covered less than 50% of the country’s territory (Table 1). These recent data were mainly derived from survey reports (85.1%), followed by natural history collections (10.7%) and literature (4.2%).

**Table 1:** Summary of Mozambique’s terrestrial mammal inventory.

	Records	Species	Cells with information	Well-known cells	Point-density mean
Whole inventory	14201	215	1014	54	109
Large mammals	5847	29	851	27	78.6
Medium mammals	2632	37	605	18	37.3
Small mammals	5722	149	458	42	76.4
Old data	8171	204	826	30	—
Recent data	6030	156	582	23	—

For the full inventory, each mammal group, and for old and recent data, are shown the number of records, species, and cells with information across the country, the number of well-known cells, and the point-density mean. The total number of cells within the 0.25° resolution country-wide grid is 1217. Old data refers to data collected before the year 2000; and recent data to data collected after the year 2000.

Mozambique has a high diversity of terrestrial ecosystems, which according to the most comprehensive synthesis on African habitats by Burgess *et al*.^26^ encompasses five biomes and, within the biomes, 13 ecoregions. Burgess *et al*.^26^ follows the definition of biome and ecoregion by Olson *et al*.^27^.

**Figure 3:**
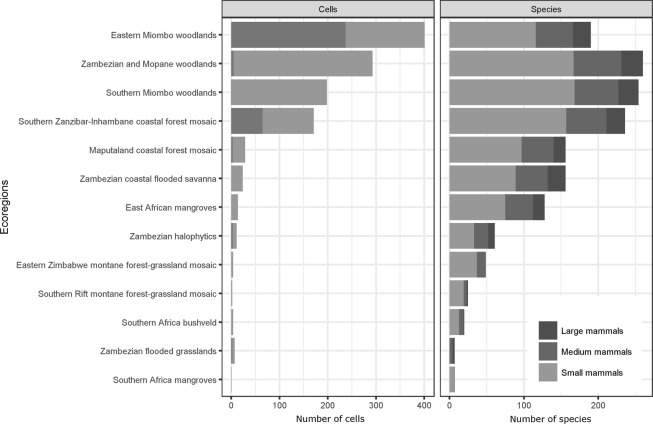
Knowledge of terrestrial mammals across Mozambique’s ecoregions. The plate “Cells” shows the number of cells at 0.25° resolution occupied by each ecoregion, using cell centroid assignment rule. Dark grey bars show the proportion of cells in each ecoregion that fall in the knowledge-gap areas. The plate “Species” indicates the number of known species in each of Mozambique’s ecoregions. The definition of the country’s ecoregions followed Burgess *et al*.^26^.

Per ecoregion, our results show a mean number of species of approximately 75.5; ranging from 4 species in the Zambezian flooded grasslands ecoregion, which covers less than 1% (0.52 +/− 0.05%) of the country, to 168 species in the Southern Miombo woodland ecoregion, which includes more than 16% of the country (16.5 +/− 0.19%; Fig. 3). The Zambezian and Mopane woodlands ecoregion also had a considerable number of species reported (167 species), as well as the Southern Zanzibar Inhambane coastal forest mosaic ecoregion (157 species). The Eastern miombo woodlands ecoregion, which covers the most extensive area within the country (33.0 + /− 0.05%), had 116 species reported.

Different sampling intensity and methods can influence data bias and gaps^28^. Overall, mammals, with such a wide range in size, can be targeted by many different sampling techniques; from the aerial census for megafauna or trapping for smaller species. To capture the potential bias in the knowledge generated by different approaches, records were classified according to the corresponding species size, considering the adult average body mass, into small mammals, medium mammals, or large mammals (see Material and methods). The most considerable portion of the records, approximately 41.2%, pertained to large mammals represented by 29 species distributed across ca. 70% of Mozambique’s territory. Large mammals were recorded in most of the ecoregions (Fig. 3). All large species were recorded in the Zambezian and Mopane woodlands ecoregion, and most were recorded in the Southern Miombo woodlands ecoregion (27 species), and the Southern Zanzibar-Inhambane coastal forest mosaic (25 species). Most of these records were obtained from survey reports (64.5%), followed by literature and natural history collections (Fig. 2).

Medium mammal data corresponds to 18.5% of the inventory, with 37 species registered in almost 50% of the territory. Medium mammals were recorded in all ecoregions, with most species documented in the Zambezian and Mopane woodlands ecoregion (35 species), and in the Southern Miombo woodlands ecoregion (32 species) (Fig. 3). Most of these records (45.3%) were obtained from survey reports, followed by literature and natural history collections.

Small mammals make up 40.3% of the records, 149 species catalogued in less than 40% of the country’s territory (Table 1). Small mammals were recorded in 12 out of 13 ecoregions in the country, with a considerable number of species recorded in the Southern Miombo woodlands ecoregion (109 species), in the Southern Zanzibar-Inhambane coastal forest mosaic (103 species), and in the Zambezian and Mopane woodlands ecoregion (103 species; Fig. 3). Most of these records were obtained from natural history collections (59.1%), followed by literature (38%; Fig. 2).

### Inventory’s record density and biases

The examination of the biases underlying primary species occurrence data can avoid erroneous interpretations of the resultant spatial patterns^12^. Therefore, firstly, record density estimates (RDE) were investigated to understand how the inventory’s records are distributed across the country. When considering data across the entire country and all mammal groups, most species occurrence records were registered in the central and southern provinces of Mozambique, with a high record density in the Maputo province (Fig. 2a; Supplementary Material – Fig. 7). The mean record density was 109 records per 0.25° resolution grid cell (Table 1). This unequal distribution of records across the country indicates spatial bias. The bias analysis performed allowed for a better understanding not only of which factors may contribute to spatial bias but also check whether spatial biases represent environmental biases as well.

To evaluate potential causes of spatial bias in the inventory, we used the following bias factors: Distance to protected areas, Distance to main roads, and Distance to province capital cities. Our results indicated an apparent over-representation of mammal records in areas close to the protected area (Supplementary Material – Fig. 8). On the other hand, areas close to roads and the main cities were under-represented (Supplementary Material – Figs. 9 and 10).

**Figure 4:**
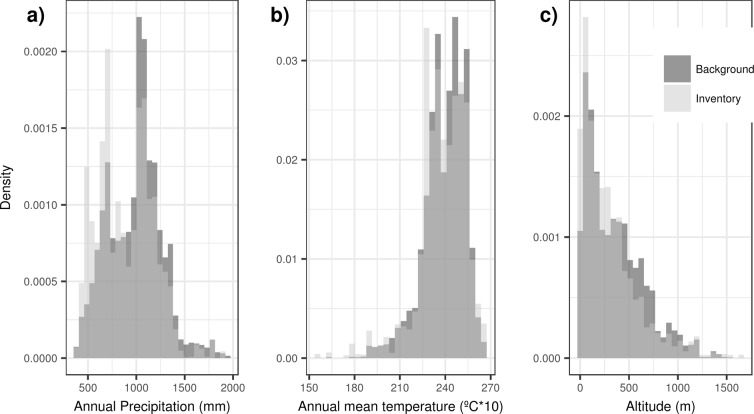
Visualization of the environmental bias in Mozambique’s terrestrial mammal inventory across three environmental variables in comparison to background data: (**a**) Annual precipitation, **(b**) Annual mean temperature, and (**c)** Altitude. These variables were compiled from the *WorldClim* database (https://www.worldclim.org/bioclim).

To assess whether the inventory’s data covered the country’s environmental conditions, the distribution of records across selected environmental variables (annual mean temperature, annual precipitation, and altitude) was compared to environmental values from points generated randomly throughout the study area (i.e. background data). Even though, based on visual inspection, the distribution of records and background data presented a similar shape for the three variables assessed (Fig. 4); our results indicate climatic bias for the three environmental variables, with significant differences between the inventory’s and the background data environmental distributions (Kolmogorov-Smirnov test, KS test, D > 0.063, p < 0.001 in all cases). In general, collecting effort was lower than expected in areas of higher annual mean temperature (>24 °C), in areas of higher annual precipitation (>1000 mm), as well as in areas with an altitude between 400 and 750 meters (Fig. 4).

With regards to the three mammal groups considered, the density maps showed parallel patterns to those found for the full inventory, i.e., high record incidence in central and southern Mozambique. (Supplementary Material – Fig. 7). Mean record densities were higher for large mammals (79 records) and small mammals (76 records) and, lower for medium mammals (37 records). Records of both large and medium mammal spatial distributions were over-represented in protected areas. Small mammal spatial distribution was slightly over-represented in protected areas and strongly over-represented near the main cities and roads.

Regarding the coverage of the country’s environmental conditions by each mammal group data, we observed, for the three groups, and with significant differences, substantial departures from background environment distributions for the three variables (KS test, D > 0.088, p < 0.001).

### Inventory completeness and well-known areas

**Figure 5:**
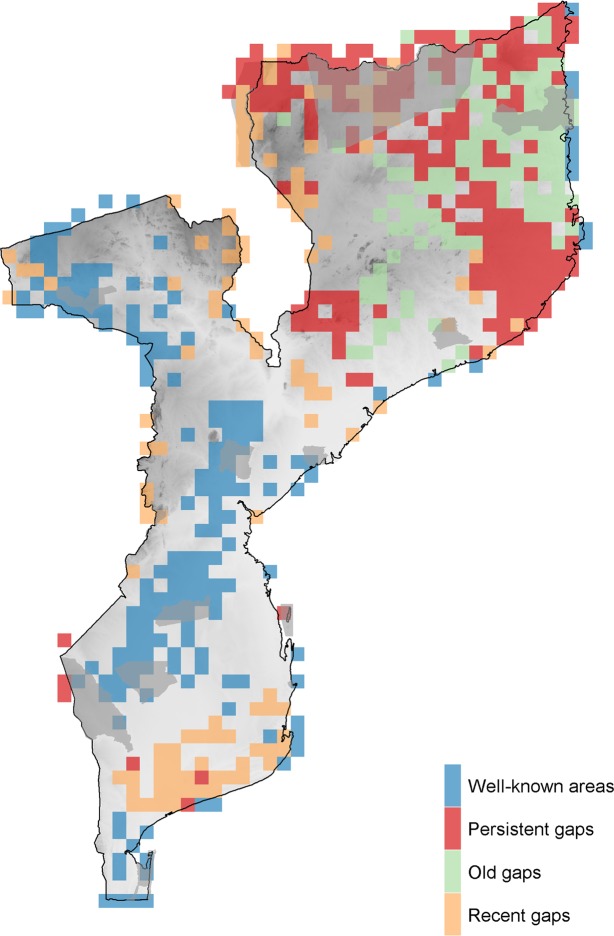
Visualization of the spatial knowledge gap areas on Mozambique’s terrestrial mammals over two periods. Knowledge gap areas result from the combination of the climatic and geographical distance to the “well-known” cells (N > 40 unique records and Inventory completeness >0.7), at 0.25° resolution. Knowledge gaps for old data and recent data were superimposed. Old data refers to data collected before the year 2000, and current data to data collected after the year 2000.

Inventory completeness for each grid cell was computed by applying the adaptation of the Chao and Jost (2012)^29^ method as proposed in Stropp *et al*.^11^ (see Material and methods). “Well-known” areas of the country were established based on the cell-wide inventory completeness. Artifactual values of inventory completeness may be obtained when inventories are based on small sample sizes^2^. Since the sample size was low for several grid cells and to define a more reliable range of completeness values, we selected a minimum sample size by inspecting for a monotonic relationship between the number of unique records and the number of species per grid cell^13^, and the relationship between the number of unique records and the values of completeness^2^. Monotonic relationships both between the number of unique records and the values of completeness and between the number of unique records and the number of species per grid cell were found for values above 40 records, approximately. Accordingly, we restricted “well-known” cells to those presenting more than 40 unique records and values of completeness above 0.7 (Supplementary Material – Fig. 11). The spatial distribution of inventory completeness at 0.25° resolution showed that 4.4% (54/1217) of cells are “well-known” (Fig. 5). Most of these “well-known” areas are located inside or near protected areas.

**Figure 6:**
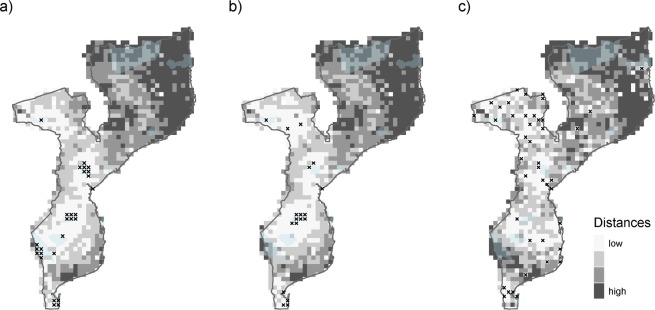
Visualization of the spatial knowledge gap areas on Mozambique’s terrestrial mammal groups: (**a**) for large mammals, (**b**) for medium mammals, and (**c**) for small mammals. Knowledge gap areas result from the combination of the climatic and geographical distance to the “well-known” cells (N > 20 unique records and Inventory completeness >0.7), at 0.25° resolution. Cells that fit the criterion of well-known grid cells for each mammal group are marked with a cross.

For the analysis per mammal group, we determined another minimum sample size by inspecting the relationship between the number of unique records and the values of completeness as previously for the full inventory. Following this criterion, and because each of these sets of records encompasses a lower record density per grid cell on average, for each mammal group cells were considered “well-known” when they presented more than 20 unique records and values of completeness above 0.7. The spatial distribution of inventory completeness showed that: 2,2% of the country’s cells are “well-known” regarding large mammals, 1,5% for medium, and 3,4% for small mammals (Table 1). Shared “well-known” cells between the three groups are located at: (i) Gorongosa National Park, (ii) Beira city and (iii) Zinave NP, near the Save River (Fig. 6).

### Knowledge gap areas

Knowledge about species occurrence and distribution, following the rationale of the principle of distance-decay of similarity in community composition, is expected to be limited in areas progressively distant from well-sampled areas^11,17^. Accordingly, here, knowledge gap areas were defined not merely as sites with low inventory completeness but also as sites that are both geographically remote and climatically different from the well-known areas^2^. To find the knowledge gap areas we determined: (i) geographical distances from all grid cells in Mozambique to the nearest “well-known” cells; (ii) climatic space based on the bioclimatic variables that retained the gradient of variation of the country’s climatic conditions; and (iii) minimum Euclidean distances among cells in the computed climatic space.

The selection of the variables that best described Mozambique’s climatic space with minimal multicollinearity was computed using a Principal Component Analysis (PCA). The first three components of the PCA accounted for 83.8% of the variability of the country’s climatic conditions. Three variables were selected to define the “bioclimatic space”, one for each component. The more representative and uncorrelated bioclimatic variables^30^ were the mean temperature of the wettest quarter, temperature seasonality, and precipitation of the driest quarter. Given the selected variables, Mozambique displays relatively homogeneous climatic conditions. Nevertheless, some sites, in northern and southern Mozambique, stand out with unique and diverse environmental conditions, such as the area of inselbergs and hills in Zambézia province, the coast of Nampula and Cabo Delgado provinces, and along the Limpopo River, Gaza province (Supplementary Material – Fig. 12).

For the whole inventory, the combination of the distance in the “bioclimatic space” with the distance to well-sampled areas showed the broadest knowledge gap area located in north-eastern Mozambique (Niassa, Cabo Delgado and Nampula provinces), and two smaller knowledge gap areas, one in western Zambézia, at the inselbergs area, and the other in the coastal Gaza province, southern Mozambique (Fig. 5). Almost 60% of the Eastern Miombo woodlands ecoregion area is within the gap areas in northern Mozambique (58.8 +/− 0.23%). More than 35% of the Southern Zanzibar-Inhambane coastal forest mosaic ecoregion is within the three identified gap areas (35.73 +/− 3.75%; Fig. 3).

For the three mammal size categories, the following knowledge-gap areas were detected: (i) one large area shared by coastal Cabo Delgado and Nampula provinces, and two narrow areas; (ii) north of the Niassa province; (iii) the inselbergs area at Zambézia province, and (iv) the coastal Gaza province. Data compiled on small mammals showed more dispersed knowledge gap areas and an additional location with lacking information was detected at the Limpopo NP, Gaza province (Fig. 6).

Considering that in Mozambique the historical data is mainly based on natural history collections originating from opportunistic or highly localised expeditions and that, in the last two decades, the sources of data were mainly reports of biodiversity surveys focussed on protected areas^7^, we assumed that different knowledge gap patterns might arise for historical and recent data. Thus, it was not only essential to understand the existing bulk of knowledge considering the full temporal coverage of the dataset (1842–2018), but also to examine whether and how sampling effort presented a different pattern temporally. For this purpose, records were grouped as: (i) “old data” if collected before the year 2000, and (ii) “recent data” if collected after the year 2000. Next, we performed a comparison of knowledge gaps for these two different temporal windows. The results revealed, as expected, different distribution patterns for old and recent data. For old data, the “well-known” areas are scattered across central and southern Mozambique close to the main cities or main roads. For recent data, “well-known” cells are all located within protected areas and Mount Namuli.

The map of the geographical and environmental distances relative to “old data” confirms the limited knowledge from northern Mozambique (Fig. 5). The analysis of “recent data” unveiled same additional low-information areas: (i) a broad area in coastal Gaza and Inhambane provinces; and scattered sites (ii) along the Chimanimani mountains, on the border with Zimbabwe, and (iii) along the left margin of the Zambezi River (Fig. 5).

## Discussion

Our study clearly shows that, in Mozambique, mammal records are not equally distributed in space. More precisely, we found that Mozambique’s mammal fauna is well-known in less than 5% of the territory, with broad areas of the country poorly sampled or not sampled at all (Fig. 5). The pattern observed from past and recent data, for all mammal groups, indicates that significant areas in northern Mozambique remain in need of further data collection, and data on large and medium mammals are over-represented in protected areas due to biases in census methods. We discuss these findings and, in light of economic growth and conservation concerns, recommend some priority areas to improve knowledge about the country’s mammal fauna.

### Inventory completeness

Our analysis exposed that the “well-known” areas in the country are related to accessibility and the existence of supporting infrastructures. For data collected before the year 2000, the “well-known” areas are located near urban areas and main roads, all in central and southern Mozambique. “Recent data” are mostly associated with protected areas across the country (including sites in north Mozambique), which are of scientific interest.

During the nineteenth and twentieth centuries, geopolitical interests in southern Africa guided European and North American scientific expeditions to preferentially survey the areas surrounding and south of the Zambezi River. These circumstances, along with the lack of transport infrastructures in the north, have meant that species in Mozambique have mostly been collected from the central and southern provinces. However, in recent years, growing political stability along with an increase in northern Mozambique’s accessibility, and political interest in biodiversity conservation have boosted monitoring effort, particularly in protected areas, which had a positive effect on inventory completeness. These events may explain the patterns detected by our analysis.

Combining the geographical and environmental survey gaps across the country, northern Mozambique emerges consistently with several knowledge gap areas. More precisely, the analysis of data collected before the year 2000 reveals a vast and contiguous area in the coastal provinces Cabo Delgado and Nampula, which falls in the Coastal forest mosaic and Eastern miombo woodlands ecoregions. A further knowledge gap area is a smaller area associated with the inselbergs and hills, the “sky island forests” (Mount Namuli, Mount Mabu, Mount Chiperone), on the western border of the Zambézia province. Increasing scientific interest in studying northern Mozambique’s inselbergs and hills, through various expeditions and surveys (e.g., Mount Mabu, Mount Inago, Mount Namuli), led to the description of new species from several taxonomic groups. From these areas with unique environmental conditions, new species of reptiles^31^, butterflies^32^, bats^5^ and plants^33^ have been recently described. These findings highlight how diverse and understudied the Afromontane forest is and support the rationale that prioritising lesser-known and environmentally unique areas for survey in Mozambique will likely locate additional records or species.

### Priorities to improve knowledge of mammal fauna from Mozambique

Increasing accessibility to primary species occurrence data allows researchers and conservationists to improve knowledge about a country’s biodiversity. The terrestrial mammal inventory used in this study relies on species-occurrence data collected between the mid-eighteen hundreds and recent years^7^. Collection dates for records associated with specimens in NHC ranged from 1845 to 2015, and scientific literature from 1985 to 2018. Data from survey reports were all published after the year 2000 (2004–2010). For the period 1990–2000, very few records of mammal occurrence were available, and very few species were reported. Mozambique experienced critical changes in this period, namely, the arrival of peace in the country in 1992, and the country’s commitment to the Convection for Biological Diversity (CBD) targets in 1994. These events influenced the amount of biodiversity data available after the year 2000, with a peak in species occurrence data from Mozambique detected in 2008, when a country-wide wildlife census was carried out^34^. However, the limited use of science for decision-making and limited knowledge about biodiversity and its potential to increase human well-being are considered indirect causes of biodiversity loss and habitat degradation in Mozambique by the Ministry of Land, Environment and Rural Development^35^.

Here, by examining similar and different knowledge gap areas in the past and recent years, we provide baseline information for terrestrial mammal species conservation and management plans.

#### Targeting unknown areas - Knowledge discovery

A large part of Mozambique remains insufficiently documented in terms of its mammal fauna (Figs. 5, 6). The knowledge-gap areas recognised in our study are mostly associated with two ecoregions (Fig. 2). The Southern Zanzibar-Inhambane coastal forest mosaic has long been described as a poorly known ecoregion regarding its mammal fauna^26,36^. For Mozambique, our study indicates that 157 mammal species were reported for this ecoregion (Fig. 2). The Eastern Miombo woodlands ecoregion, the largest in Mozambique, is also poorly known regarding mammal occurrence. When compared with Southern Miombo, located in southern and central areas of the country, Eastern Miombo woodlands present a lower number of species (116 species) than the former (168 species). Henceforward, true species richness may be higher than presently estimated, especially in northern Mozambique.

Although the lack of accessibility and infrastructure in the north were partially resolved, the last two decades of studies on biodiversity were not sufficient to change this pattern of less knowledge for this region. Consequently, there is an urgent need to prioritise these areas in future field surveys. It is worth noting that a significant part of the knowledge gap falls within the Niassa NR, which reportedly supports the major remaining concentrations of carnivores and ungulates in Mozambique^34,37^. Despite the recent surveys in Niassa NR, none investigated small mammal diversity.

#### Targeting the lesser-known mammal groups

Overall, less information has been gathered on small and inconspicuous fauna, because recent surveys in Mozambique are almost exclusively based on aerial counts, which mostly detect the conspicuous medium and large species^7,34,38^. Accordingly, spatial distributions of large and medium mammal records were over-represented in protected areas.

When multiple census methods were used in recent surveys, we observed a shift from gap to well-known areas. This scenario occurred in 9% of the country, mainly due to broad surveys taken in Quirimbas NP and Mount Namuli^7^ (Fig. 5), and shows that more complete inventories depend on the inclusion of varied census methods to register the presence of mammal groups, which are highly variable in terms of size, behaviour and habitat preferences.

For small mammals, well-known areas are scattered across the country and data is biased towards the main cities and roads (Figs. 4, 6). Some protected areas present an evident lack of knowledge for this group, with wide gaps in Limpopo NP, Niassa NR, and small areas in Maputo Special Reserve. Large and medium mammals are well-known groups in the protected areas of southern and central Mozambique. However, in the north, there is still a lack of knowledge of these groups in Niassa NR and Quirimbas NP. Increasing the surveys’ taxonomic extent inside the protected areas is a resource-efficient way towards the achievement of international commitments such as the CBD’s Aichi targets^39,40^, namely to protect the complete range of biodiversity present in areas of importance for biodiversity (CBD’s Strategic Objective B - Target 11).

#### Targeting known areas – Spatiotemporal studies

Our work pinpoints poorly known environmentally different areas while recognising similar environmental areas that were regularly visited over time. These areas correspond to 14% of the country, mostly across the protected areas (Fig. 6). As examples, Gorongosa NP and Zinave NP are well-known areas for the three mammal groups. It is essential to continue to collect data from these sites because this will enhance the collective knowledge on biodiversity through retrospective and comparative studies. The existence of historical and recent data enables the evaluation of changes in biodiversity and the analysis of drivers of distribution changes^41^, or the selection of areas of interest for species reintroduction^42^. For instance, by comparing data from a recent survey and an expedition in the mid-1920s, the authors of a study in the Ethiopian highlands were able to document shifts associated with climate change in the former ranges of five small mammal species over approximately 90 years^41^.

Our study also detected that, for some areas of Mozambique, the potential of spatiotemporal studies could be lost. Over the last two decades, some unique climatic areas in central and southern Mozambique emerged as less surveyed. Notably, there was a broad knowledge gap area on the coast of Gaza province (Fig. 6), which was recently described as having undergone extensive habitat loss^4^. Although this finding may be conjectural, an effort should be made to avoid the discontinuity of monitoring effort in this area, thus preserving the potential for spatiotemporal studies.

#### Improving knowledge - data mobilisation

The usefulness of primary species-occurrence data to improve biodiversity knowledge can be fully realised by increasing the availability of useful quality data. The previous work performed in the compilation, digitalisation, cleaning and validation of the inventory on Mozambique’s mammal diversity^7^ was pivotal to identify survey priorities and to improve knowledge. Nonetheless, it should be noted that the identified knowledge gap areas may not solely reflect the lack of collection effort but may also correspond to existing information not included or not easily accessible. Thus, besides the enhancement of sampling effort, improved access to further biodiversity data, along with the digitisation of natural history collections and better overall dissemination of recent internal research will address more complex biological questions and will provide the foundation for the effective conservation of biodiversity. This strategy could be an effective way to rapidly close gaps and reduce data biases in poorly documented and research-neglected countries^40,43^.

### Biodiversity data

Filling biodiversity knowledge gaps requires prioritisation of efforts not only to compile additional data but also to evaluate and enhance the quality of the data already available and to make it accessible. Works from Ballesteros-Meija *et al*.^44^, Stropp *et al*.^11^, Marques *et al*.^45^, and Queirós Neves *et al*.^7^ are recent examples for African countries.

Many developing countries are understudied and present a severe lack of species-occurrence data^43^, which is worsened by the poor dissemination of these research data. Furthermore, most data on African mammal collections (~95%) stored in the GBIF platform are provided by European and North American institutions.

Thus, improving knowledge of the biodiversity of poorly documented countries can only be achieved by allocating resources to expand and promote national and international initiatives, with a strong emphasis on capacity-building of national and local institutions. Positive progress has been made in this direction. For example, Biodiversity Information for Development (BID) is a multi-year programme funded by the European Union and led by GBIF to increase the amount of biodiversity information available in the nations of sub-Saharan Africa, the Caribbean and the Pacific (https://bid.gbif.org). Of the 23 projects financed thus far, Mozambique is participating in an “African Insect Atlas”, which aims to unleash the potential of insects in conservation and sustainability research (https://www.gbif.org/project/82632/african-insect-atlas).

## Conclusion

It is most important to fill knowledge gaps on species occurrence and distribution, especially if the aim is to expand the taxonomic extent of conservation planning. A conservation planning based on accurate species occurrence data is even more crucial in countries where high poverty rates, sporadic armed conflicts, intensive exploration of natural resources and extreme weather events accrue. Deprived of reasonable information regarding species occurrence, it is unmanageable to concentrate efforts to preserve diversity and guide conservation actions.

Based on primary species occurrence data, which span the years from 1845 to today, we identified provinces in Mozambique that are poorly documented regarding terrestrial mammal fauna (e.g., Niassa, Cabo Delgado, Nampula and Tete). These provinces are vastly explored for oil, coal, hydrocarbons and minerals^46^, presenting severe challenges for biodiversity conservation. Moreover, the high population growth observed in the northern provinces is associated with agricultural development and habitat degradation^47,48^. Given that habitat loss is a leading cause of biodiversity decline, there is an urgency to study and survey the provinces identified in this study since some economic activities, such as mine-exploration and plantation forestry, without proper impact studies may lead to irreversible biodiversity loss^49,50^.

Finally, the assessment of the knowledge gaps from primary species occurrence data showed to be a powerful strategy to generate information that is essential to species conservation and management plan, particularly for understudied countries.

## Material and Methods

### Study area

The Republic of Mozambique, located on the Indian coast of Southeast Africa, holds a vast territory of more than 800,000 square kilometres (Fig. 1). The climate is generally tropical and dry, but temperature and precipitation are highly variable throughout the country^51^. The country is considered vulnerable to natural disasters and currently presents an increasing incidence of flood and drought events^52^. The centre of the country, recently impacted by cyclone Idai, is more prone to floods and tropical cyclones, followed by the south and the north^52^.

A large part of Mozambique’s topography is characterised by flat terrain extending from coastal lowlands in the east to mountain ranges in the west (Fig. 1). The country has a high diversity of terrestrial ecosystems, which are, following the African ecoregions defined in Burgess *et al*.^26^, represented by 13 ecoregions. The Eastern miombo woodlands ecoregion covers a large area of the country, mostly in north Mozambique, followed by the Zambezian and Mopane woodlands ecoregion, in central and southern Mozambique, the Southern Miombo woodlands ecoregion, in central Mozambique, and the Southern Zanzibar-Inhambane coastal forest ecoregion, along most of the coast of the country.

### Terrestrial mammal inventory

We used primary species occurrence data on terrestrial mammals from Mozambique compiled and validated^7^. The authors considered the following sources of species occurrence data: (i) natural history collections, mainly data from the Global Biodiversity Information Facility portal (GBIF), (ii) survey reports on the main protected areas and other areas of ecological interest; and (iii) literature - including the first published species checklist of Mozambican mammals^53^. Species occurrence data compiled were validated by thorough data quality assessment and improvements, namely data cleaning, georeferencing and taxonomic update. For details refer to^7^. The compilation generated a species checklist of mammals reported from Mozambique (n = 217). Here, we analysed the underlying data that supports this species checklist, holding a total of 14981 records (Table 1). Approximately, 34.2% of the records were reported from surveys, 33.1% from natural history collections, and the remaining 32.7% from the literature.

The species occurrence data were reduced to unique records to avoid duplication of information. Accordingly, each unique record represents a pool of registries from a single species collected in the same locality, by the same collector, on the same day. Localities of occurrence were considered identical when latitude and longitude (with 2-digit precision) coincided.

### Data treatment

Records were aggregated to a 0.25° spatial resolution grid, and the total number of grid cells across the country was 1217. This spatial resolution was selected by assessing the balance between the accuracy of aggregated data versus the loss of spatial resolution, as in^13,23^ (Supplementary Material – Fig. 13 shows three different data resolutions). All analyses and mapping in this study were carried out in the R programming environment^54^.

To obtain general information on the proportion of each ecoregion cover across the country and the respectively assigned species richness, we extracted the terrestrial ecoregion (and associated biome) at the centroid of each 0.25° cell by overlaying the grid on the ecoregions map^26^. Thus, each grid cell was attributed to a terrestrial ecoregion. A sensitivity analysis using other assignment rules was performed (Supplementary Material Fig. 14). Even though we observed a slight variation in the number of cells assigned to each ecoregion, which is higher for the ecoregions with smaller cover in the country, the results suggested that the “Cell centroid method” is robust for our analysis, with little variation in the final results (Supplementary Material Figs. 14, 16). The ecoregions and biomes considered for Mozambique in this study followed the work on African terrestrial biomes, ecoregions and habitats by Burgess *et al*.^26^. We obtained spatial data on ecoregions from the World-Wide Foundation Terrestrials Ecoregions of the World dataset (WWF; www.worldwildlife.org/publications/terrestrial-ecoregions-of-the-world). Also, records were organised according to mammal size taking into account adult average body mass: (i) small mammals with an average body mass of fewer than five kilograms^55^; (ii) large mammals with an average body mass over 25 kilograms, and (iii) medium mammals with a body size between the previous classes. Most data on species adult average body mass were retrieved from the species traits database, PanTHERIA^56^.

#### Record density and bias analysis

Record density estimates (RDE) were determined through point pattern analysis, as proposed in^20^. Geographical coordinates of the localities of occurrence represented the “points” in the analysis. First, we calculated RDE as the average number of localities per 0.25° grid cell and, subsequently, we created density maps using an isotropic Gaussian kernel.

The magnitude of spatial bias in the records was defined by splitting each bias factor into four intervals, using the Fisher algorithm, based on the range of the measured distances to the factor analysed^57^. The Fisher algorithm selects classes in which both similar values are grouped, and the difference between classes area is maximized^20^. Hence, “interval 1” represented the area where distances to the bias factor are smallest, while in “interval 4” distances were highest.

The spatial variables considered as potential bias factors were: (i) distance to protected areas; (ii) distance to main roads; and (iii) distance to province capital cities. The bias was quantified for each interval following Kadmon *et al*.^20,58^:

1.1 $$Bia{s}_{i}=\frac{{n}_{i}-{p}_{i}\,N}{\sqrt{{p}_{i}\,(1-{p}_{i})N}}$$

where ${n}_{i}$ is the number of localities of occurrence within a specified interval (*i*), *N* is the total number of localities of occurrence in the database, and ${p}_{i}$ is the independent probability that a given locality of occurrence will lie within an interval – the Kadmon’s bias index.

The Eq. (1.1) is derived from a normal approximation to the binomial distribution. Thus, since the value of the index is distributed like a standard normal variable (Z), the bias becomes statistically significant for values greater than 1.64 (at α = 0.05). Hence, for each interval of distances to the bias factors, bias values greater than 1.64 characterise over-represented areas, that is areas with more localities of occurrence than expected from a random sampling design. On the other hand, bias values less than –1.64 show under-sampled areas. The Kadmon’s bias index (*p*) was estimated by generating the same number of random replacement points (i.e. localities of occurrence) as in the inventory and calculating the fraction of points on each interval. The formulation of random points and the estimation of the bias index were repeated 100 times, and bootstrap statistics and confidence intervals were calculated.

Subsequently, we assessed whether the localities of occurrence of the inventory’s unique records covered the country’s environmental conditions randomly. The environmental bias factors analysed were: (i) annual mean temperature, (ii) annual precipitation; and (iii) altitude. These three variables were compiled from the *Worldclim* database^30^. The bias was evaluated by comparing the distribution of the localities of occurrence to the distribution of the background environment for each variable. The background environment was based on randomly generated points (with replacement) across the study area. Next, for both sets of points, we extracted the corresponding values of the selected bioclimatic variables. Those values were then compared using the Kolmogorov-Smirnov test (KS). The KS assesses the null hypothesis that the frequency distribution of two samples is drawn from the same continuous distribution^59^. The KS *D-*statistic was used as an index of congruence between the localities of occurrence and the background environment^60^. The KS was computed using the ks.test function (R package: *dgof)*.

#### Spatial distribution of inventory completeness and “well known” cells

Inventory completeness was computed for each grid cell. The method applied was proposed by Stropp *et al*.^11^, and is an adaptation of the Chao and Jost (2012)^29^ method; given by:

1.2 $${C}_{i}=\frac{{f}_{1i}}{{n}_{i}}\times [\frac{({n}_{i}-1){f}_{1i}}{({n}_{i}-1){f}_{1i}+2{f}_{2i}}]$$

where *C*_*i*_ = estimated inventory completeness; *n*_*i*_*, f*_*1i*_*, f*_*2i*_, = number of observations/specimens, singletons and doubletons found in grid cell *i*. *C*_*i*_ ranges from zero to one, with one indicating a complete inventory.

Two additional approaches to calculating inventory completeness were tested: the inventory completeness based on Sousa-Baena *et al*. (2014) and species accumulation curves as in Yang *et al*. (2013). However, the adapted Chao and Jost (2012) method was the only one that resulted in a monotonic relationship between inventory completeness and the number of records (Supplementary Material – Fig. 11).

We analysed the cell-wide inventory completeness to define the “well-known” areas of the country. Since the sample size was small for several grid cells, we obtained artefactual high values of completeness. To define a more reliable range of completeness values we selected a minimum sample size looking for a monotonic relationship between the number of unique records and the number of species per grid cell^13^, and between the number of unique records and the values of completeness^2^.

#### Knowledge-gap areas

The knowledge-gap areas were defined as areas with insufficient sampling and that are geographically distant and climatically different from the well-known areas. Diverse studies followed this rationale^2,23,24^. Thus, firstly, we determined the geographical distances from all grid cells in Mozambique to the nearest “well known” cells.

Secondly, we selected the bioclimatic variables that retained the gradient of variation of the country’s climatic conditions. Climatic space was characterised in terms of the most representative and uncorrelated variables of the 19 bioclimatic variables of the *WorldClim* database^30^ for Mozambique. *WorldClim*’s variables are based on the average monthly temperature and rainfall registered from 1970 to 2000. The selection of the variables that best described the climatic space with minimal multicollinearity was computed using a Principal Component Analysis (PCA). We selected first the number of principal components required to account for 80% of the total explained variance. Then, we chose bioclimatic variables that contributed most to each principal component dimension with minimal correlation to one another.

Thirdly, we determined the environmental distances to the well-known cells by calculating the minimum Euclidean distances among the country’s cells in the computed climatic space. Next, the geographical and environmental distances were scaled from 0 to 10 and multiplied to produce a map of “space and environment uniqueness” creating a parallel view of the environmental distances from well-known cells.

Finally, we considered as knowledge-gap areas the sites of “space and environment uniqueness” that showed several adjacent cells with distance values above the third quantile in the range of distances to the “well-known” areas.

Further, we performed a comparison of knowledge gaps for two different temporal windows. Records were grouped as: (i) “old data” if collected before the year 2000, and (ii) “recent data” if collected after the year 2000. To inspect changes in the spatial patterns of the knowledge gaps between the two temporal windows, we superimposed the gaps obtained with data collected before the year 2000 and the following two decades. Additionally, to identify the ecoregions within knowledge gap areas and to determine their proportion of cover, we intersected the knowledge gap areas with the ecoregions map and extracted for each ecoregion the number of cells with their centroid within the gap areas.

## Supplementary information

Supplementary figures

## Data Availability

The datasets generated during and/or analysed during the current study are available in the “Mendeley data” repository, DOI: 0.17632/9bkjv99bdk.1.
