# Supplementary material for: Mapping Knowledge Gaps of Mozambique’s Terrestrial Mammals
Source: Sci Rep. 2019 Dec 3;9:18184. doi: 10.1038/s41598-019-54590-4 (PMC6890665; doi:10.1038/s41598-019-54590-4)
Supplement: Supplementary file 1 — Supplementary figures [file 41598_2019_54590_MOESM1_ESM.pdf]

**SUPPLEMENTARY MATERIAL:**

# MAPPING KNOWLEDGE GAPS OF MOZAMBIQUE'S TERRESTRIAL MAMMALS

Isabel Queirós Neves, Maria da Luz Mathias, and Cristiane Bastos-Silveira

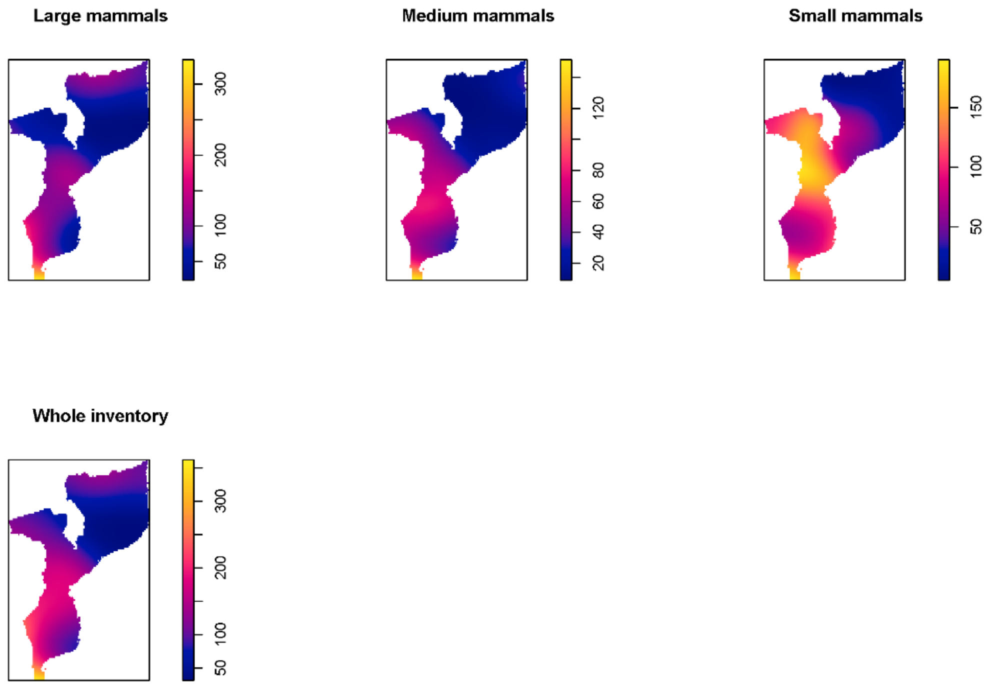


**Fig. 7** - Visualization of the records' density patterns estimated based on the isotropic Gaussian kernel of the entire inventory, and per mammal group.

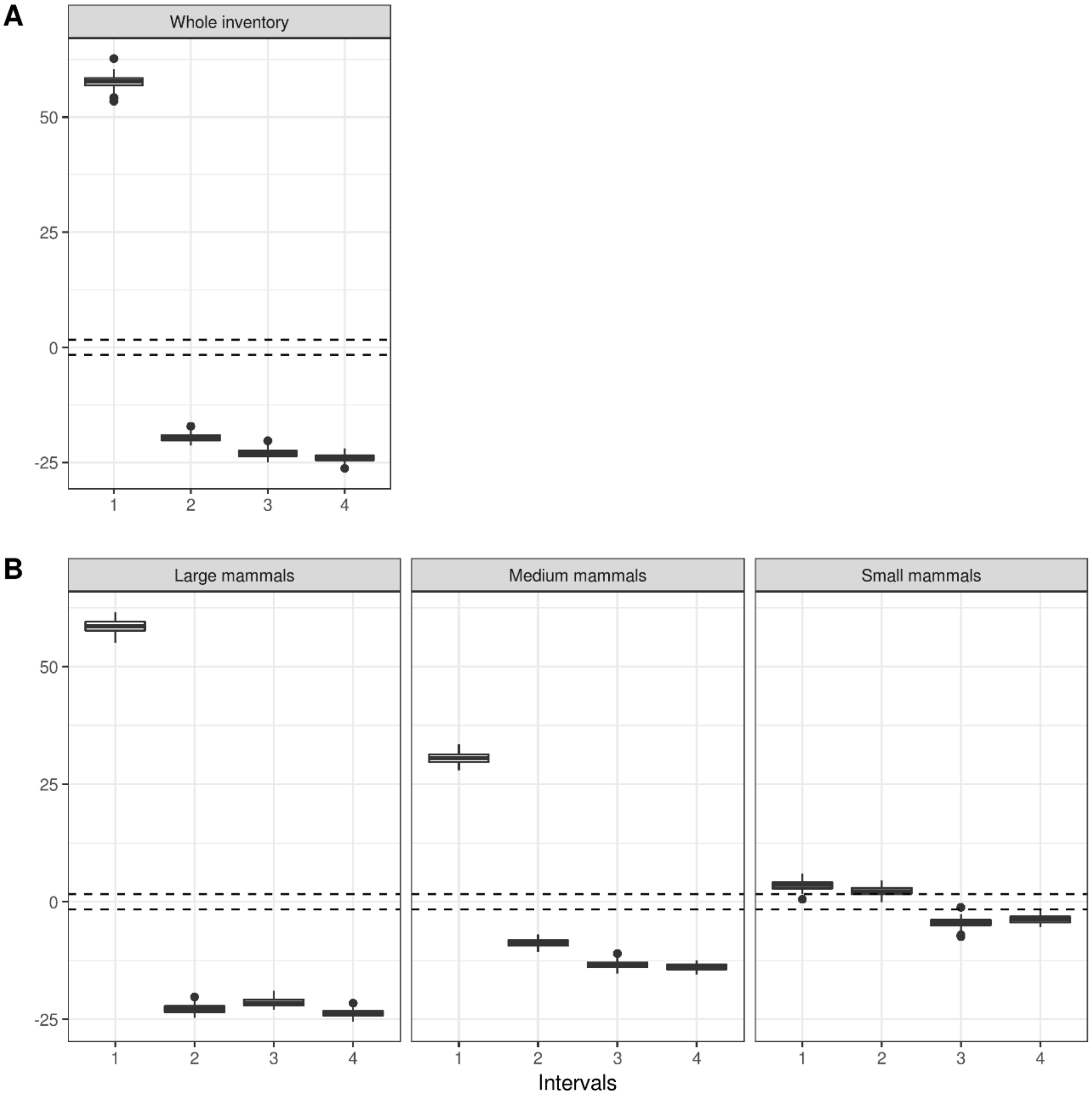


**Fig. 8** - Bias estimates to "distance to protected areas" for A) the full inventory, and B) "mammal size". Bias estimates were calculated following Kadmon et al. (2004) for each distance interval from "interval 1" for short distances to "interval 4" for the largest distances. The dashed lines mark the range of values where no bias is expected (between -1.64 and 1.64). If the boxplots are within the dashed lines, then the number of localities is as expected from a random sampling scheme. Boxplots above and below this area are an over or under-representation of records' localities of occurrence in that interval, respectively.

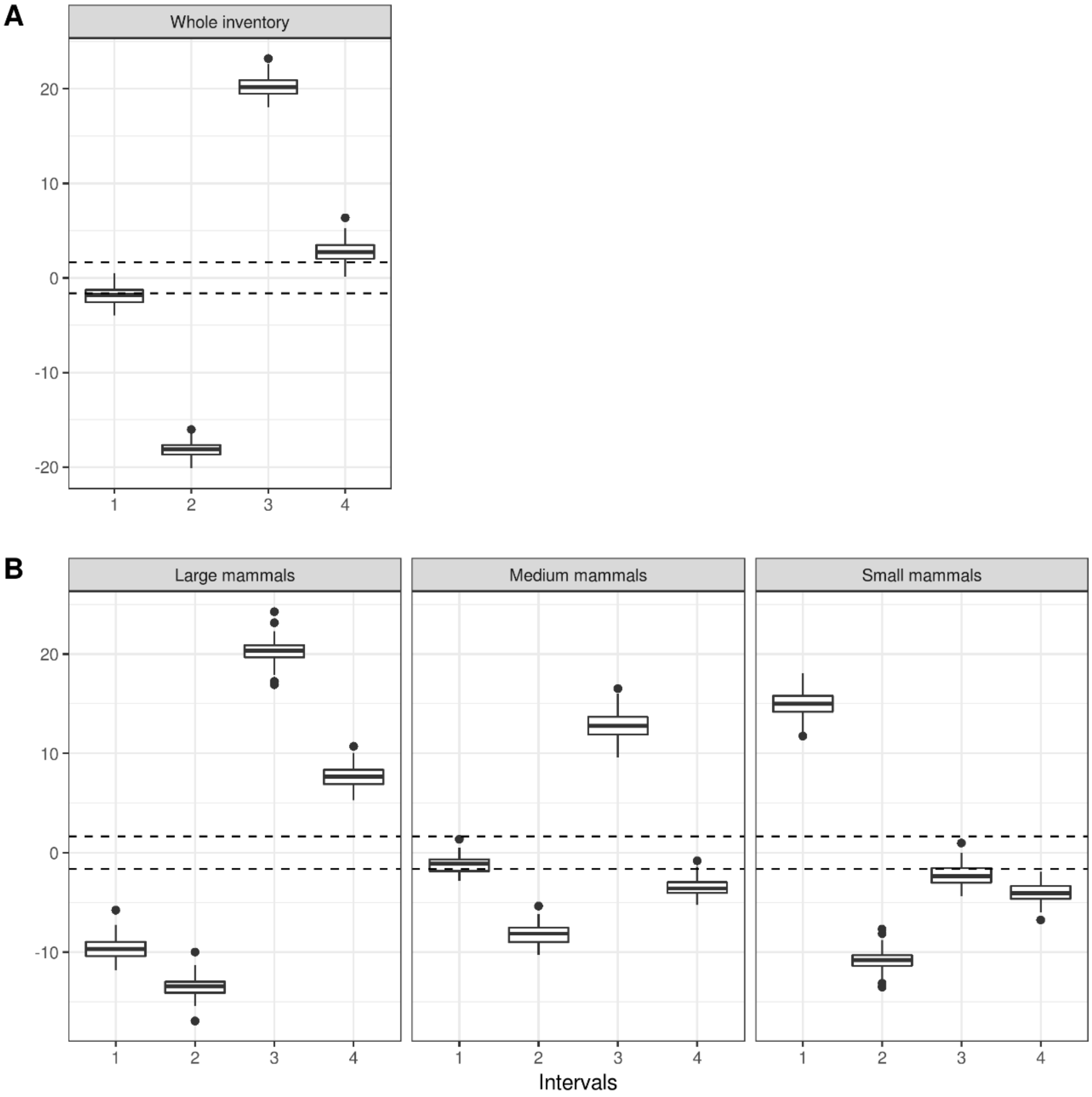


**Fig. 9** - Bias estimates to “distance to main cities” for A) the full inventory, and B) the mammal groups. Bias estimates were calculated following Kadmon et al. (2004) for each distance interval from “interval 1” for short distances to “interval 4” for the largest distances. The dashed lines mark the range of values where no bias is expected (between -1.64 and 1.64). If the boxplots are within the dashed lines, then the number of localities is as expected from a random sampling scheme. Boxplots above and below this area are an over- or under-representation of records’ localities of occurrence in that interval, respectively. The cities included in this factor are the capitals of the provinces.

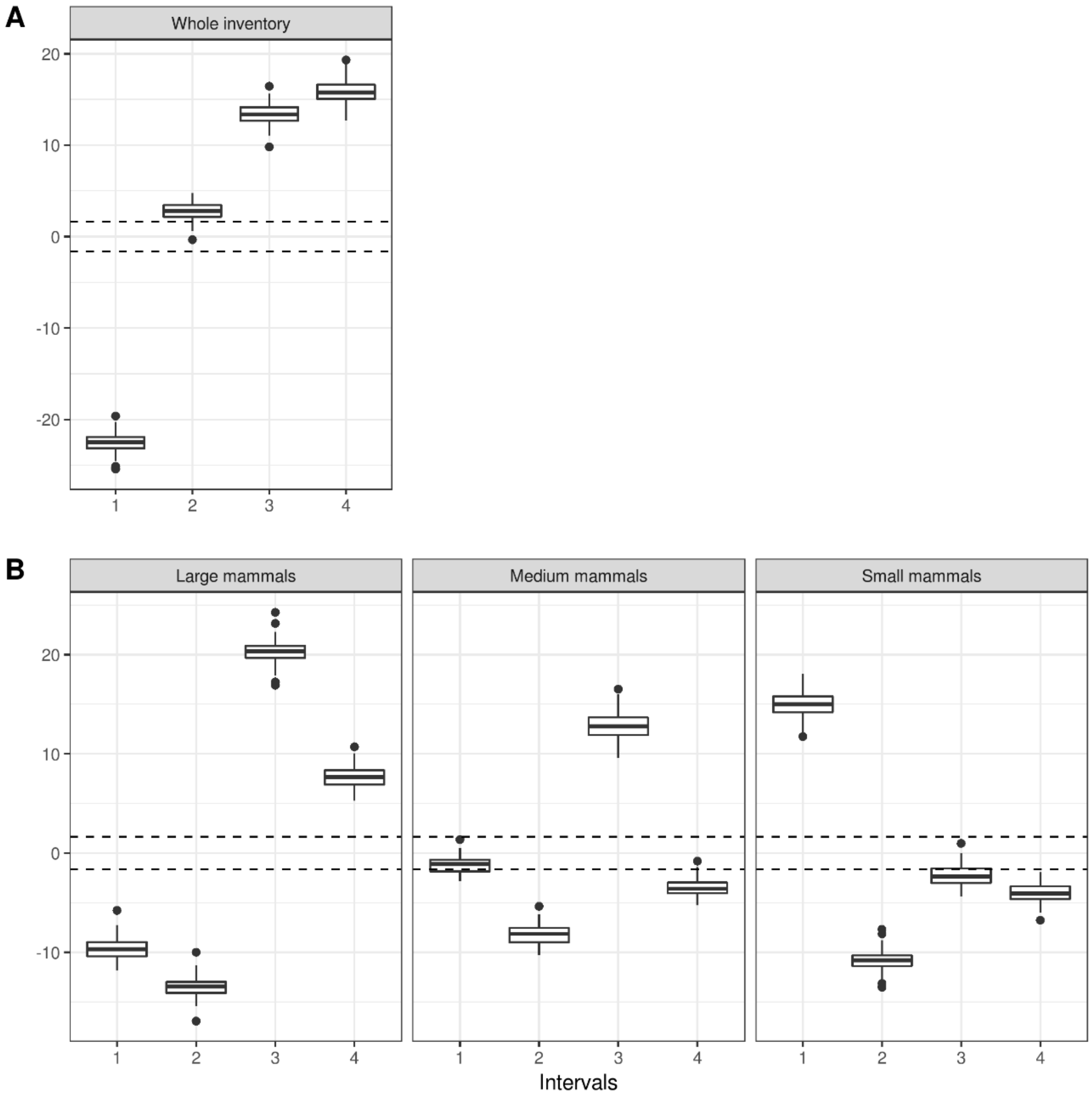


**Fig. 10** - Bias estimates to “distance to main primary roads” for A) the full inventory, and B) the mammal groups. Bias estimates were calculated following Kadmon et al. (2004) for each distance interval from “interval 1” for short distances to “interval 4” for the largest distances. The dashed lines mark the range of values where no bias is expected (between -1.64 and 1.64). If the boxplots are within the dashed lines, then the number of localities is as expected from a random sampling scheme. Boxplots above and below this area are an over- or under-representation of records’ localities of occurrence in that interval, respectively**.**

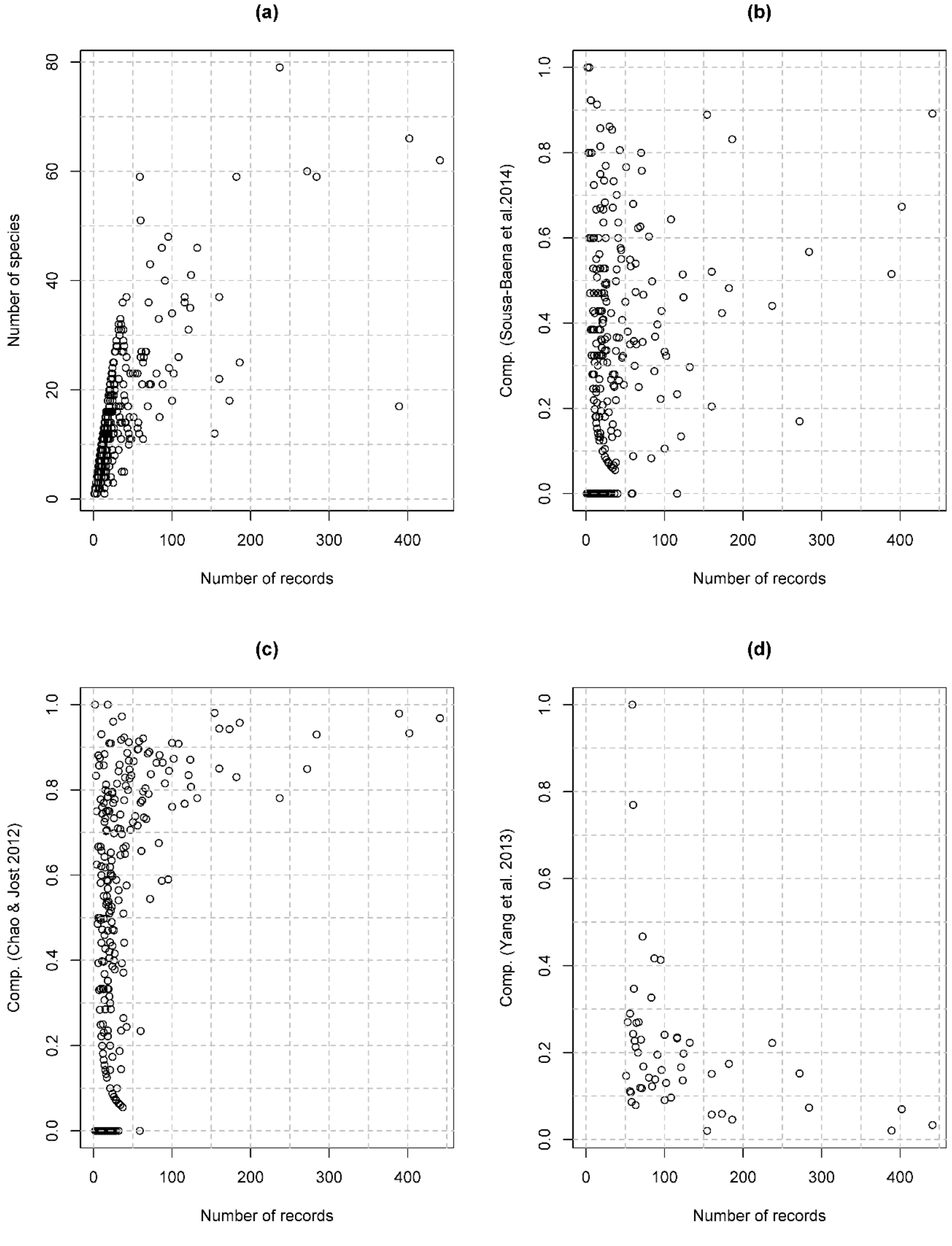


**Fig. 11** - Relationship between the number of unique records (i.e. unique combination of date, location of collection and species name) per grid cell and (a) number of species, and between the number of unique records and the estimates of inventory completeness ("Comp.") obtained according to according to the three methods tested in this study: (b) Sousa-Baena et al. (2014), (c) Chao & Jost (2012) and (d) the curvilinearity of species accumulation curves according to Yang et al. (2013).

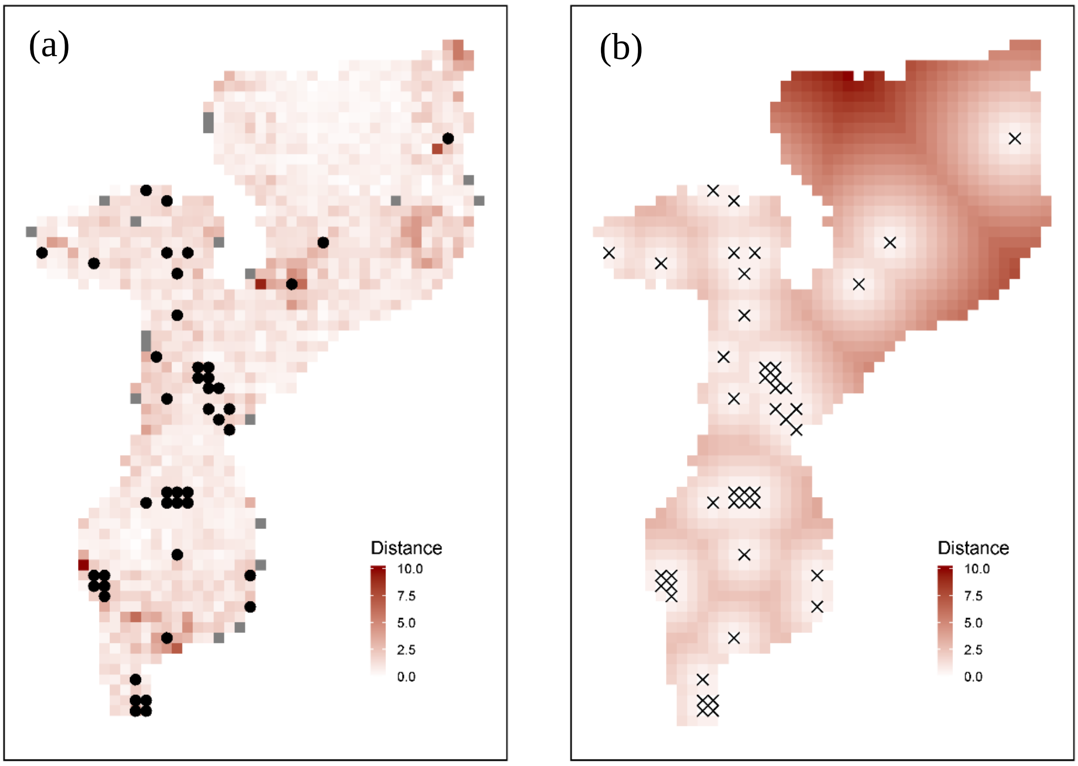


**Fig. 12** – Spatial visualisation of the distance measured throughout the country's cells at 0.25ºresolution in (a) the bioclimatic space between the cells, and (b) from the well-known cells regarding terrestrial mammal sampling in Mozambique. The country's climatic space was defined by the following bioclimatic variables: Mean temperature of the wettest quarter, Temperature seasonality, and Precipitation of the driest quarter. Bioclimatic variables were obtained from the *WorldClim* database (Fick and Hijmans, 2017). Cells that fit the criterion of well-known grid cells are marked with a dark point in (a) and with a cross in (b).

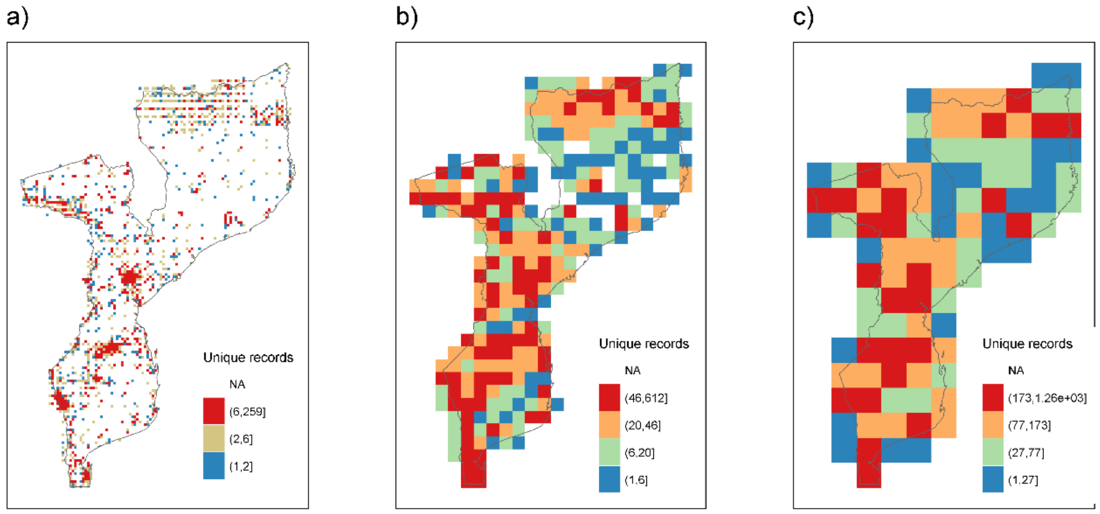


**Fig.13** – Visualization of the number of unique records across Mozambique based on grids of different spatial resolutions: a) 0.1º; b) 0.25º; c) 1º.

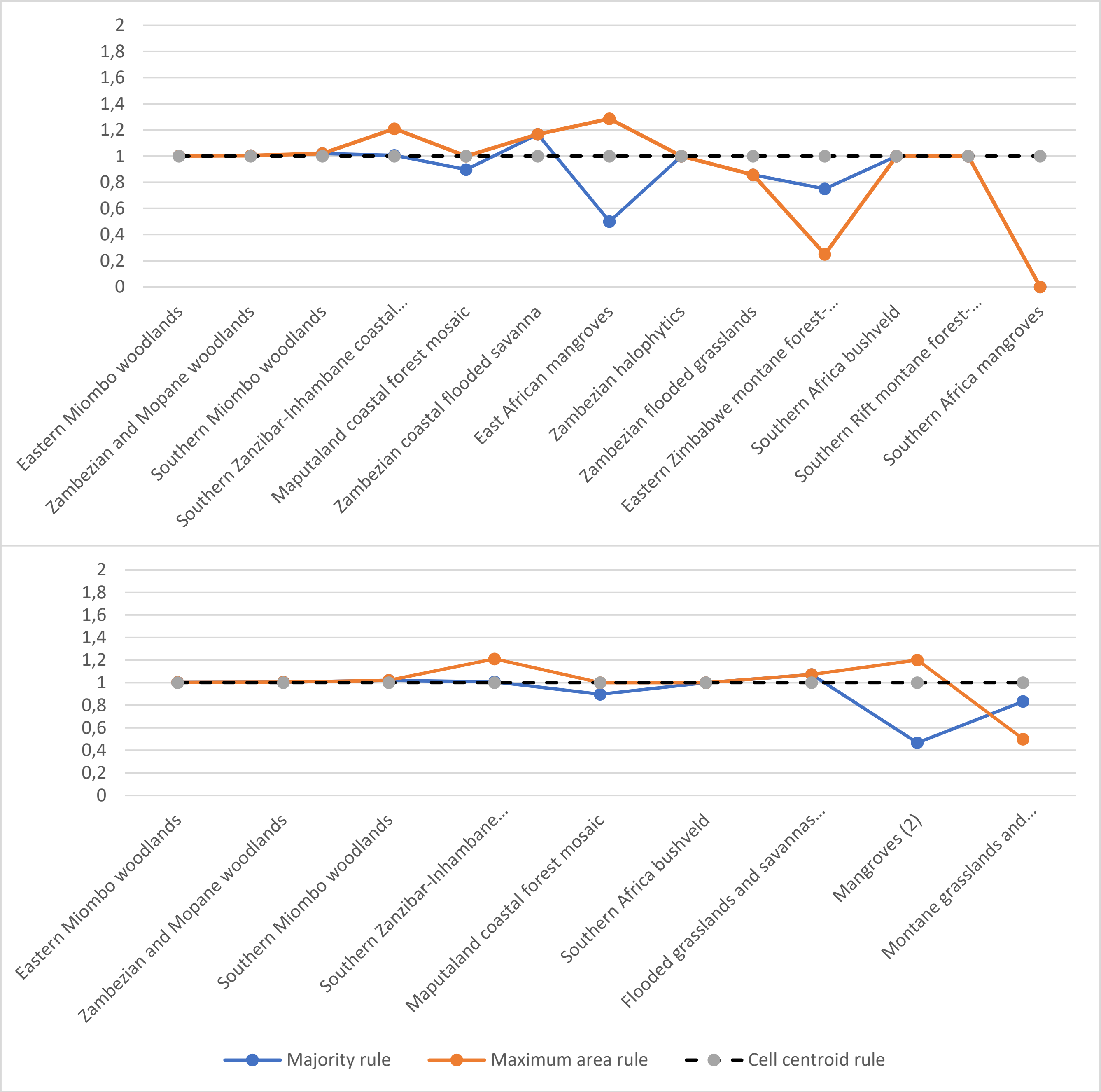


**Fig 14**. Sensitivity analysis for different assignment methods of ecoregions to grid cells: (a) for each ecoregion with cover in Mozambique; and (b) for each ecoregion with cover in Mozambique, with the less extensive ecoregions aggregated in biomes. Sensitivity was measured as the ratio between the proportion of cells within a specific ecoregion using another assignment methods and the assignment method used in our study. For this study, we assign the terrestrial ecoregions (and associated biome) by overlaying ecoregions map onto the country's grid, and the ecoregion that overlaps each cell centroid is assigned to the cells ("Cell centroid method"). Two assignment methods were tested and compared to "Cell centroid rule": "Maximum area rule" and "Majority rule". In the "Maximum area rule" the largest ecoregion is assigned to the cells; and in the "Majority rule" the ecoregion that overlaps by at least 50 per cent is assigned to the cell, or, when multiple ecoregions overlap a cell, the largest overlapping area must be greater than the area in the cell that is not covered by any ecoregion. Values of sensitivity close to 1 reveal a result that is robust independently of the method chosen for assigning cells to ecoregions. The ecoregions and biomes considered for Mozambique in this study followed a last comprehensive assessment of African terrestrial biomes, ecoregions and habitats (Burgess *et al.*, 2004). Spatial data was downloaded from WWF Terrestrials Ecoregions of the World dataset (www.worldwildlife.org/publications/terrestrial-ecoregions-of-the-world).

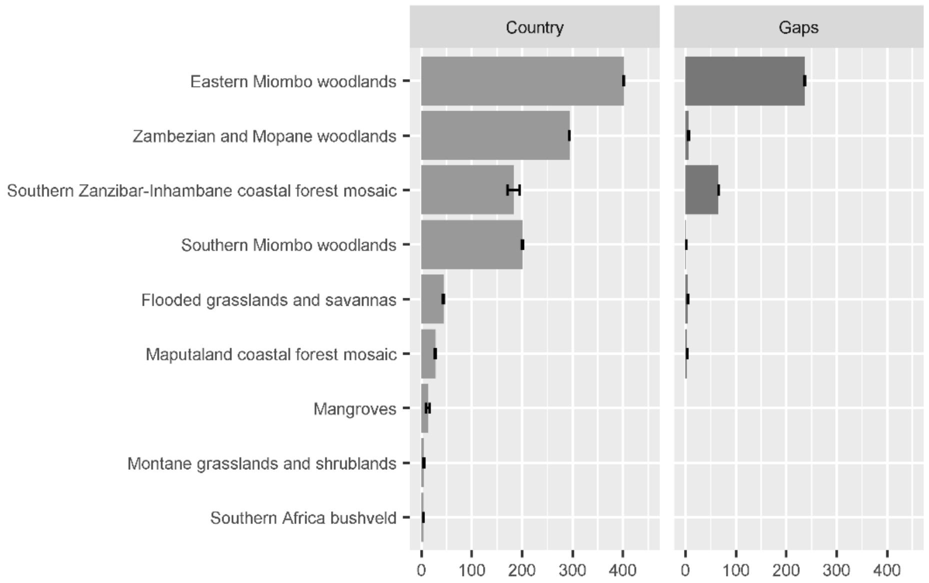


**Fig. 15.** Barplot showing the number of country's cells and the number of gap cells at 0.25º resolution occupied by each ecoregion with different polygon-cell assignment rules.
